# *LncFZD6* initiates Wnt/β-catenin and liver TIC self-renewal through BRG1-mediated FZD6 transcriptional activation

**DOI:** 10.1038/s41388-018-0203-6

**Published:** 2018-03-14

**Authors:** Zhenzhen Chen, Yanfeng Gao, Lintong Yao, Yating Liu, Lan Huang, Zhongyi Yan, Wenshan Zhao, Pingping Zhu, Haibo Weng

**Affiliations:** 1 School of Life Sciences, Zhengzhou University, Zhengzhou, 450001 China; 2 Collaborative Innovation Center of New Drug Research and Safety Evaluation, Zhengzhou, 450001 Henan Province China; 3 The First Affiliated Hospital, Zhengzhou University, Zhengzhou, 450052 China

**Keywords:** Extracellular signalling molecules, Cancer stem cells

## Abstract

Liver tumor-initiating cells (TICs), the drivers for liver tumorigenesis, accounts for liver tumor initiation, metastasis, drug resistance and relapse. Wnt/β-catenin signaling pathway emerges as a critical modulator in liver TIC self-renewal. However, the molecular mechanism of Wnt/β-catenin initiation in liver tumorigenesis and liver TICs is still elusive. Here, we examined the expression pattern of 10 Wnt receptors (FZD1–FZD10), and found only FZD6 is overexpressed along with liver tumorigenesis. What’s more, a divergent lncRNA of FZD6, termed *lncFZD6*, is also highly expressed in liver cancer and liver TICs. *LncFZD6* drives liver TIC self-renewal and tumor initiation capacity through FZD6-dependent manner. *LncFZD6* interacts with BRG1-embedded SWI/SNF complex and recruits it to *FZD6* promoter, and thus drives the transcriptional initiation of FZD6 by chromatin remodeling. WNT5A, a ligand of FZD6, is highly expressed in liver non-TICs and drives the self-renewal of liver TICs through *lncFZD6*-BRG1-FZD6-dependent manner. Through FZD6 transcriptional regulation in cis, *lncFZD6* activates Wnt/β-catenin signaling in liver TICs. *LncFZD6*-BRG1-Wnt5A/β-catenin pathway can serve as a target for liver TIC elimination. Altogether, *lncFZD6* promotes Wnt/β-catenin activation and liver TIC self-renewal through BRG1-dependent FZD6 expression.

## Introduction

Liver cancer is one of the most serious cancers in the world. Liver cancer contains two common tumor types, hepatocellular carcinoma (HCC) and cholangiocarcinoma (CC). Heterogeneity is a major characteristic of liver cancer and largely increases the difficulty for clinical elimination [1]. Actually, there are several kinds of cells in liver samples. Liver tumor-initiating cells (TICs), a small subset cells within liver tumor bulk, account for liver tumor initiation, metastasis and relapse [2, 3]. Liver TICs have the capacities of self-renewal and differentiation [4]. They generate various cell types and can propagate to refuel their population during tumorigenesis. According to these capacities, some functional assays were developed: sphere formation for self-renewal, side population for drug resistance, xenograft for propagation, gradient xenograft for tumor initiation and so on [5–7]. Recently, more and more surface markers of liver TICs have been discovered, including CD133, CD13, CD24, EPCAM and so on [8–10]. Other than surface marker, several transcription factors are also involved in liver TIC self-renewal, including c-Myc, Oct4, Zic2, Sox4 and so on [11, 12]. However, the biological characteristics of liver TICs remain elusive.

Several signaling pathways participate in liver TIC self-renewal, including Wnt/β-catenin signaling, Notch signaling and Hedgehog signaling [13–15]. Among these pathways, the role of Wnt/β-catenin signaling in liver TICs is widely explored [16]. Wnt/β-catenin signaling plays a central role in liver TIC self-renewal, and its activation is under precise regulation [17–19]. In Wnt-OFF cells, β-catenin interacts with APC, Axin2, β-TrCP to form APC complex, which triggered β-catenin ubiquitinoylation and subsequent degradation [20]. Upon Wnt ligands bind to their receptors (FZD1-FZD10), Wnt/β-catenin signaling is activated. The conformational changes of FZD receptors induce the disruption of APC complex, releasing β-catenin from degradation complex, followed by nuclear translocation of β-catenin [21]. β-Catenin interacts with T-cell factor/lymphoid enhancer-binding factor (TCF/LEF) complex in cell nuclei to drive target gene expression [22]. Although the regulatory mechanisms of Wnt/β-catenin pathway have been deeply explored, how it is activated in liver tumorigenesis and liver TICs is largely unknown.

FZD receptors, encoded by “frizzled” gene family, are the receptors for Wnt molecules, and responsible for Wnt/β-catenin activation upon Wnt binding [23]. There are 10 FZD receptors in human cells, FZD1-FZD10. Here we found FZD6 is highly expressed in liver cancer and liver TICs. FZD6 contains a signal peptide and a cysteine-rich domain in N-terminal and seven transmembrane domains [24]. However, unlike other FZD proteins, FCD6 does not contain a PDZ domain-binding motif in C-terminal [25]. The function of FZD6 in Wnt/beta-catenin activation is controversial. FZD6 acts as a negative regulator of Wnt3a induced Wnt/β-catenin activation when co-expressed with FZD1 [26]. However, Wnt4 can bind to FZD6 and induce the activation of Wnt/β-catenin/LEF/TCF signaling cascades, through a yet unknown mechanism [27]. The physiological and pathological role of FZD6 in Wnt/β-catenin activation is still unclear.

Long noncoding RNAs (LncRNAs) are RNA transcripts with longer than 200 nucleotides in length and no protein-coding potential [28]. Recent studies reveals lncRNAs are important mediators in many biological processes, including tumorigenesis [29]. In tumors, lncRNAs participate in tumor initiation, metastasis, colony formation, energy metabolism and so on [30–33]. LncRNAs exert their roles through transcriptional regulation or post-transcriptional regulation. LncRNAs interact with chromatin remodeling complex and participate the transcription of neighbor or distant genes in cis or in trans [34]. For post-transcriptional regulation, lncRNAs combine with core components of signaling pathways, and change the stability or activity of associated proteins [29, 31]. Here, we found *lncFZD6* and FZD6 are highly expressed in liver cancer cells and liver TICs. *LncFZD6* and FZD6 are required for liver TIC self-renewal. *LncFZD6* interacts and recruits BRG1 to *FZD6* promoter to initiate transcription. *LncFZD6*-BRG1-FZD6 signaling pathway can be a target for liver TIC elimination.

## Results

### FZD6 is highly expressed in liver cancer and liver TICs

Liver TICs account for liver initiation, invasion, metastasis and relapse. The self-renewal capacity of liver TIC self-renewal is under precise regulation. Using unbiased screening for lncRNAs, we found that a lncRNA termed *lncSox4* participated in liver TIC self-renewal and liver tumorigenesis. *LncSox4* recruits Stat3 to the promoter of Sox4, and thus drives Sox4 expression and liver TIC self-renewal [12]. Wnt/β-catenin signaling is the most important signaling in liver TIC self-renewal. However, the initiation of Wnt/β-catenin signaling is largely unknown.

**Fig. 1:**
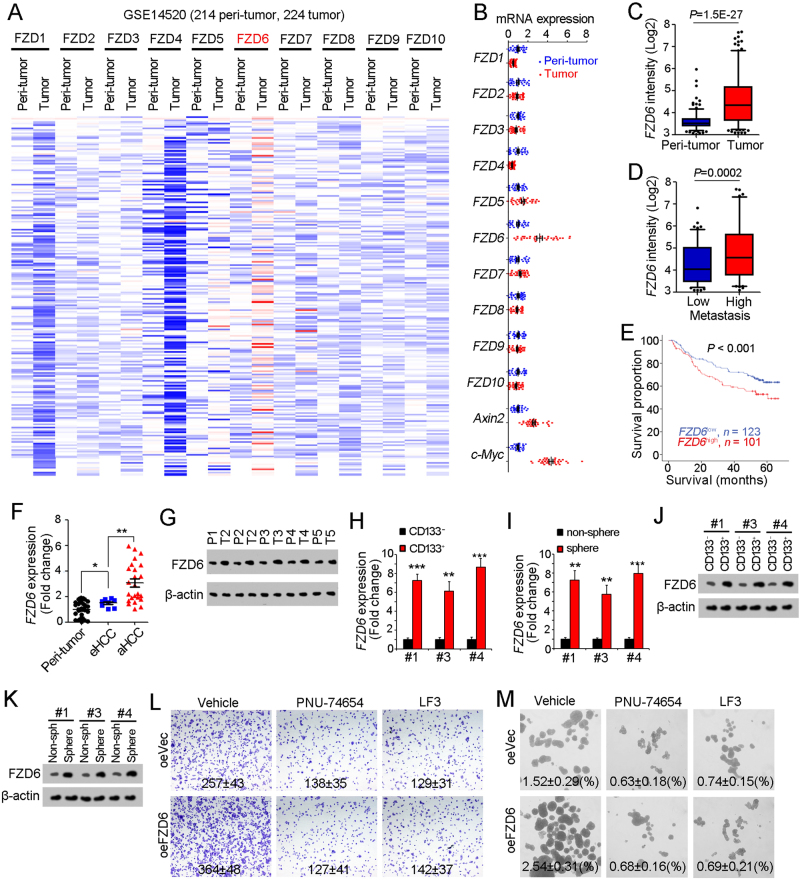
High expression of FZD6 in liver cancer and liver TICs. **a** Heatmap of Wnt receptors (FZD1-FZD10) in 214 peri-tumor samples and 224 tumor samples derived from GSE14520. The average expression levels in peri-tumor samples were defined as 1. FZD6 is highly expressed during liver tumorigenesis. **b** Thirty-two peri-tumor samples and 32 HCC samples were examined for expression levels of the indicated genes. Ten FZD members and two Wnt/β-catenin target genes (Axin2, c-Myc) were analyzed. **c**, **d** Samples were divided into two groups according to sample origin **c** or metastasis **d**, and FZD6 intensity were analyzed and shown as box and whisker plot. Boxes indicate interquartile ranges (IQR); upper and lower edges are the 75th and 25th percentiles. Horizontal lines within boxes are median intensity. Whiskers are 5th and 95th percentiles. **e** Samples were divided into two groups according to FZD6 expression levels, followed by Kaplan–Meier survival analysis. **f**, **g** Peri-tumor, early hepatocellular carcinoma (eHCC) and advanced hepatocellular carcinoma (aHCC) samples were collected, and FZD6 expression levels were detected using real-time PCR **f** and western blot **g**. **h**, **i** FZD6 mRNA expression levels were detected using CD133 enriched liver TICs **h** or oncospheres **i**. FZD6 expression in non-TICs was served as control. **j**, **k** liver TICs **j** and oncospheres **k** were enriched and FZD6 protein levels were examined with western blot. **l**, **m** FZD6 overexpressed (oeFZD6) and control (oeVec) cells were treated with β-catenin/TCF inhibitors (PNU-74654, LF3), and tumor invasion **l** and self-renewal capacities **m** were examined. Typical images and calculated numbers (or ratios) were shown. For **b**,** f**,** h**,** i,** data were shown as means ± s.d. For **c**, **d**, data are shown as box and whisker plot. *P < 0.05; **P < 0.01; ***P < 0.001 by two-tailed Student’s *t*-test. Data are representative of three independent experiments

The activation of Wnt/β-catenin needs the combination of Wnt ligands and Wnt receptors. The expression levels of Wnt receptors play a fundamental role in Wnt/β-catenin activation. Here, we examined the expression levels of Wnt receptor (FZD1–FZD10) using online available dataset (GSE14520) [35, 36]. Of the then FZD receptors, FZD6 is highly expressed along with liver tumorigenesis (Fig. 1a). We confirmed the high expression of FZD6 and Wnt/β-catenin target genes (Axin2 and c-Myc) along with liver tumorigenesis using clinical samples (Fig. 1b). FZD6 is also related to liver tumorigenesis, tumor metastasis and prognosis (Figs. 1c-e). To further examine the expression pattern of FZD6, we collected liver cancer samples, examined the expression levels of FZD6 with real-time PCR and western blot, and confirmed the increased FZD6 expression both at mRNA levels and protein levels (Figs. 1f, g).

Considering the critical role of Wnt/β-catenin in liver TIC self-renewal, we then detect FZD6 expression levels in liver TICs. We enriched liver TICs through two strategies: sorting with surface marker CD133 and sphere formation assay. We found both in CD133^+^ liver TICs and oncospheres, FZD6 is highly expressed (Figs. 1h, i). We then confirmed the real-time PCR results with western blot, and also found FZD6 is highly expressed in liver TICs at protein levels (Figs. 1j, k). Considering the role of FZD6 in Wnt/β-catenin activation is elusive, we overexpressed FZD6 in β-catenin inhibited cells, and found impaired influence on tumor invasion and self-renewal (Figs. 1l, m), indicating that FZD6 drives liver tumor invasion and liver TIC self-renewal through Wnt/β-catenin signaling. Altogether, FZD6 is highly expressed in liver cancer and liver TICs.

### High expression of *lncFZD6* in liver cancer and liver TICs

**Fig. 2:**
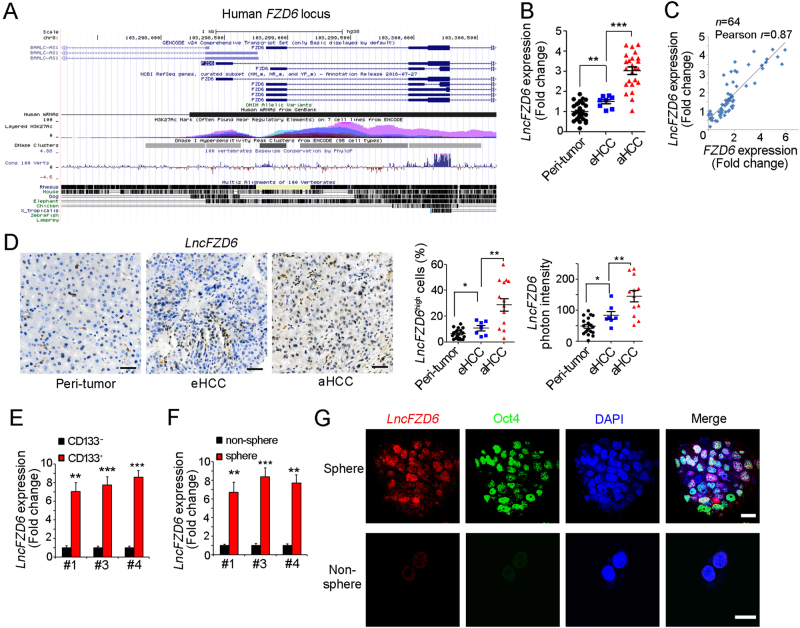
*LncFZD6* was highly expressed in liver cancer and liver TICs. **a** Human FZD6 locus was shown using UCSC Genome Browser. There is a divergent lncRNA (BAALC-AS1, here was termed as *lncFZD6*) in near from FZD6 locus. **b** *LncFZD6* expression levels were detected using real-time PCR. The average of *lncFZD6* expression levels in peri-tumors were served as control. **c** The correlation between *FZD6* and *lncFZD6* expression levels. The fold changes of *FZD6* and *lncFZD6* in the sample tumor samples were used for scatter diagram, and Pearson correlation coefficient was calculated. Sixty-four samples were detected. **d** In situ hybridization (ISH) of *lncFZD6* in peri-tumor, early hepatocellular carcinoma (eHCC) and advanced hepatocellular carcinoma (aHCC) samples. Typical photos were shown in left panels and calculated ratios were shown in right panels. **e** CD133^+^ liver TICs and CD133^-^ liver non-TICs were enriched with FACS, followed by real-time PCR analysis for *lncFZD6* expression. *LncFZD6* expression levels in non-TICs were controls. Three samples were shown. **f** Sphere formation assays were performed, and *lncFZD6* expression levels were detected with real-time PCR. **g** Fluorescence in situ hybridization of *lncFZD6* using spheres and non-spheres. Oct4 was a positive control. Scale bars,** d**, 50 μm; **g**, 10 μm. Data were shown as means ± s.d. *P < 0.05; **P < 0.01; ***P < 0.001 by two-tailed Student’s *t-*test. Data are representative of three independent experiments

We next wanted to explore the molecular mechanism of FZD6 expression. First, we silenced several TIC-related molecules (including c-MYC, AXIN2, SOX2, TP53, STAT3, ZIC2, NOTCH2, SOX4, TCF7 and OCT4), and found knockdown of these genes had no changes of *FZD6* mRNA expression, but FZD6 knockdown induced impaired c-MYC expression (Supplementary Figure 1A, B). These results indicated that FZD6 was a “upstream” factor and cannot be regulated by these key TIC-related molecules. To explore the mechanism of FZD6 expression, we focused on FZD6 gene locus, and found lncRNA ABBLC-ASC (hereafter termed *lncFZD6*) is a divergent lncRNA of FZD6. The distance of their transcription start sites is <1 kb (Fig. 2a). *LncFZD6* is also highly expressed in HCC samples, especially in advanced HCC samples (Fig. 2b). Interestingly, *lncFZD6* is co-expressed with FZD6, with a high Pearson correlation coefficient (0.87) (Fig. 2c). We then detected *lncFZD6* expression with in situ hybridization (ISH). The staining results showed increased *lncFZD6* expression levels in liver tumor, especially in advanced liver tumor (Fig. 2d). Of note, only a small subset of tumor cells showed high expression of *lncFZD6*, even in advanced samples (Fig. 2d).

As the high expression of FZD6 in liver TICs and the correlation between FZD6 and *lncFZD6* expression pattern, we next examined the expression of *lncFZD6* in liver TICs. We obtained CD133^+^ liver TICs and oncospheres, examined *lncFZD6* expression, and found increased *lncFZD6* transcripts in liver TICs (Figs. 2e, f). We then performed fluorescence in situ hybridization (FISH) with oncospheres and non-spheres, confirming the high expression levels of *lncFZD6* in oncospheres (Fig. 2g). Altogether, *lncFZD6* is highly expressed in liver cancer and liver TICs.

### *LncFZD6* promotes the self-renewal of liver TIC

**Fig. 3:**
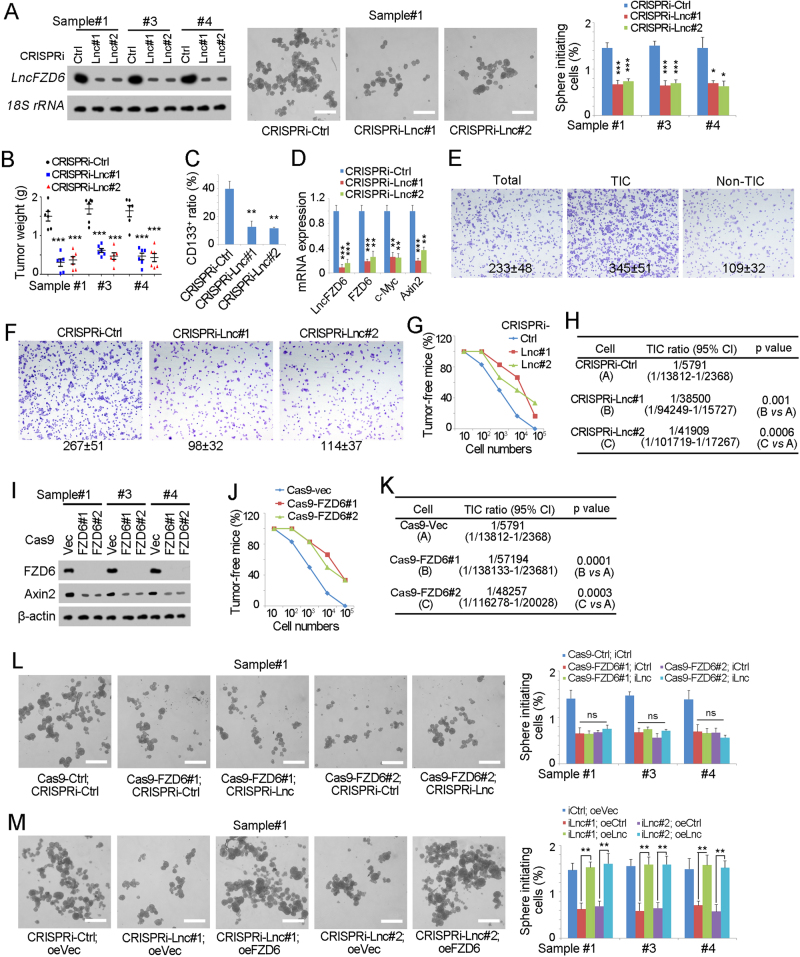
*LncFZD6* was required for liver TIC self-renewal. **a** *LncFZD6* silenced cells were established (left panels) with CRISPRi and sphere formation assays were performed. The efficiency of *lncFZD6* knockdown was detected by northern blot (left panels). Typical pictures were shown in middle panels and calculated ratios were shown in right panels. **b** In all, 1 × 10^6^ *lncFZD6* silenced or control TICs were injected into BALB/c nude mice. One month later, mice were sacrificed and the weight of tumors were detected and shown as scatter diagram. Six mice were used for each assay. **c** The indicated tumors were obtained and stained with CD133 antibody, followed by FACS examination. The ratios of CD133^+^ cells were shown. *n* = 6 for each group. **d** Total RNA were extracted from the indicated tumors, and expression levels of the indicated transcripts were examined by real-time PCR. **e** CD133^+^ TICs and CD133^-^ non-TICs were enriched and tumor invasion capacity was examined. Typical images and calculated ratios were shown. **f** *lncFZD6* silenced or control cells were used for transwell assay, and typical images were shown. **g**, **h** In all, 10, 1 × 10^2^, 1 × 10^3^, 1 × 10^4^ and 1 × 10^5^ *lncFZD6* silenced cells and control cells were injected into BALB/c nude mice for 3 months’ tumor formation. Three months later, tumor formation was observed and the ratios of tumor-free mice were calculated **g**. TIC ratios were analyzed using extreme limiting dilution analysis **h**. 95% CI 95% confidence interval of the estimation, vs versus. **i** *FZD6* knockout cells were generated with CRISPR/Cas9 approach, and knockout efficiency was determined with western blot. β-Actin served as a loading control. **j**, **k** In all, 10, 1 × 10^2^, 1 × 10^3^, 1 × 10^4^ and 1 × 10^5^ *FZD6* knockout cells and control cells were injected into BALB/c nude mice and tumor formation was analyzed as **g**, **h**. **l** *LncFZD6* was silenced in *FZD6* knockout cells or control cells, followed by sphere formation assays. Typical pictures were shown in left panels and calculated sphere-initiating ratios were shown in right panels. **m** FZD6 expression was rescued in *lncFZD6* depleted TICs, followed by oncosphere formation. Typical pictures were shown in left panels and TIC ratios were shown in right panels. Scale bars, 500 μm. Data were shown as means ± s.d. *P < 0.05; **P < 0.01; ***P < 0.001 by two-tailed Student’s* t*-test; ns not significant. Data are representative of three independent experiments

We then explored the role of *lncFZD6* in liver TIC self-renewal. We depleted *lncFZD6* expression with CRISPRi strategy, and found impaired sphere formation capacity upon *lncFZD6* depletion, indicating the critical role of *lncFZD6* in liver TIC self-renewal (Fig. 3a). We also examined the role of *lncFZD6* in liver TIC proliferation and apoptosis, and found *lncFZD6* mainly affected the proliferation of liver TICs (Supplementary Figure 1C, D). We then injected 1 × 10^6^ *lncFZD6* silenced or control TICs into BALB/c nude mice, and found impaired tumor propagation of *lncFZD6* depleted cells (Fig. 3b). What’s more, decreased TIC ratios and impaired FZD6 were found upon *lncFZD6* depletion, confirming the critical role of *lncFZD6* in live TIC self-renewal and FZD6 expression (Figs. 3c, d). Using transwell assay, we also confirmed the enhanced invasion capacity of liver TICs and FZD6 drives liver tumor invasion (Figs. 3e, f). In vivo experiments also validated the critical of *lncFZD6* in tumor invasion (Supplementary Figure 1E). To further detect the role of *lncFZD6* in tumor initiation, we injected 10, 1 × 10^2^, 1 × 10^3^, 1 × 10^4^ and 1 × 10^5^ *lncFZD6* silenced cells into BALB/c nude mice, followed by 3 months’ tumor formation. *LncFZD6* silenced cells showed impaired tumor initiation capacities, confirming the critical role of *lncFZD6* in liver TIC self-renewal (Figs. 3g, h).

We then wanted to know whether *lncFZD6* participated in liver TIC self-renewal through FZD6. We established FZD6 knockout cells using CRISPR/Cas9 approach, and validated FZD6 knockout with western blot (Fig. 3i). FZD6 deleted cells showed impaired tumor initiation capacity (Figs. 3j, k), indicating the critical role of FZD6 in liver TICs. We then silenced *lncFZD6* in FZD6 knockout cells, and found *lncFZD6* knockdown had no obvious influence on liver TIC self-renewal, suggesting that *lncFZD6* exert its role through FZD6-dependent manner (Fig. 3l). FZD6 rescue in *lncFZD6* depleted cells also rescued the impaired self-renewal capacity of liver TICs, confirming that *lncFZD6* exerted its role through FZD6 (Fig. 3m). In conclusion, *lncFZD6* drove liver TIC self-renewal through FZD6.

### *LncFZD6* recruits BRG1 to *FZD6* promoter

**Fig. 4:**
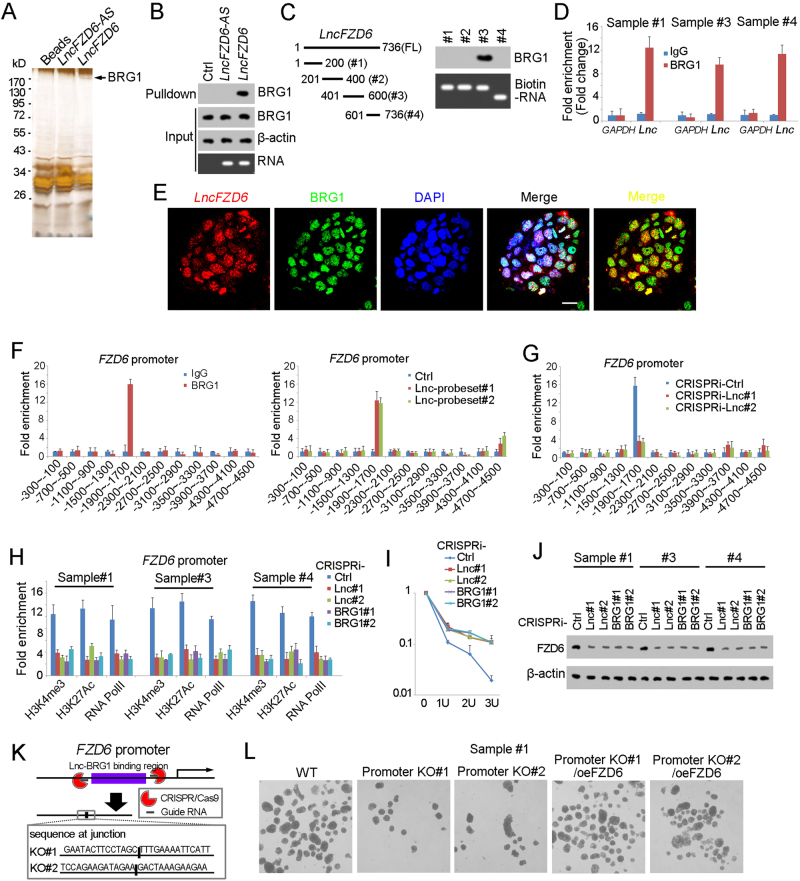
*LncFZD6* recruits BRG1 to *FZD6* promoter. **a** RNA pulldown as performed and the specific band of *lncFZD6* was identified as BRG1 with mass spectrum. **b** The interaction between *lncFZD6* and BRG1 was confirmed by western blot. β-Actin served as a loading control. **c** *LncFZD6* truncates were constructed (left panels), followed by RNA pulldown and western blot (right panels). **d** RNA immunoprecipitation (RIP) assay were performed with oncospheres derived from clinical samples, and enrichment of *lncFZD6* and GAPDH were examined with real-time PCR. IgG is an isotype antibody control. Data were shown as means ± s.d. **e** Double FISH assays showed the colocalization of *lncFZD6* and BRG1. Scale bars, 10 μm. **f** ChIP and ChIRP assays were performed with BRG1 antibody and *lncFZD6* probes, respectively, followed by *FZD6* promoter detection with real-time PCR. BRG1 and *lncFZD6* bind to the same region of *FZD6* promoter. **g** *LncFZD6* silenced and control cells were used for BRG1 ChIP assay and impaired combination between BRG1 and *FZD6* promoter was found in *lncFZD6* silenced cells. **h** *LncFZD6* and BRG1 silenced cells were used for ChIP assays with H3K4me3, H3K27Ac and RNA polymerase II. *FZD6* promoter enrichment was analyzed by real-time PCR. **i** DNase sensibility assays were performed using *lncFZD6* and BRG1 silenced cells. **j** *LncFZD6* and BRG1 silenced cells were lyzed for FZD6 western blot. β-Actin served as a loading control. **k** The *lncFZD6*-BRG1 binding region on *FZD6* promoter was deleted using CRISPR/Cas9 approach (upper panel), and confirmed by DNA sequencing (lower panel). **l** Sphere formation assays were performed using *FZD6* promoter deleted cells (promoter KO) and FZD6 rescued cells (promoter KO/oeFZD6) cells, and typical images were shown. Data are representative of three independent experiments

We then explored the molecular mechanism of *lncFZD6* in liver TIC self-renewal. We performed RNA pulldown assay and found a specific band in *lncFZD6* enrichment, which was identified as BRG1 with mass spectrum, indicating the interaction between *lncFZD6* and BRG1 (Fig. 4a). RNA pulldown and western blot also confirmed the interaction between *lncFZD6* and BRG1 (Fig. 4b). We also constructed truncates and examined the interaction between these truncates and BRG1. The third region of *lncFZD6* (#3) was identified as the binding site with BRG1 (Fig. 4c). We also detected the physiological interaction between BRG1 and *lncFZD6* using RNA immunoprecipitation (RIP) (Fig. 4d). Finally, we performed double FISH and found colocalization between *lncFZD6* and BRG1 in oncospheres (Fig. 4e). Altogether, *lncFZD6* interacts with BRG1 in liver TICs.

BRG1 is a core component of SWI/SNF complex and plays an essential role in chromatin remodeling and transcriptional regulation. So we next explored the role of *lncFZD6* and BRG1 in FZD6 transcription regulation. First, we performed chromosome immunoprecipitation (ChIP) and Chromatin isolation by RNA purification (ChIRP) assays, followed by *FZD6* promoter examination, and found both BRG1 and *lncFZD6* bound to *FZD6* promoter. Of note, BRG1 and *lncFZD6* interacted with the same region of *FZD6* promoter (Fig. 4f). We then examined the interaction between BRG1 and *FZD6* promoter in *lncFZD6* silenced cells, and found impaired binding of BRG1 to *FZD6* promoter upon *lncFZD6* depletion, indicating the critical role of *lncFZD6* in the combination between BRG1 and *FZD6* promoter (Fig. 4g).

We then detected the role of BRG1 and *lncFZD6* in FZD6 transcriptional activities. Considering the critical role of BRG1 in histone modification, we detected the histone modification levels in *lncFZD6* and BRG1 silenced cells, and found impaired histone modification upon *lncFZD6* and BRG1 depletion, indicating the involvement of *lncFZD6* and BRG1 in *FZD6* promoter activation (Fig. 4h). We also performed DNase sensibility assay, and found enhanced resistance to DNase digestion, indicating the inactivation state of *FZD6* promoter upon *lncFZD6* and BRG1 depletion (Fig. 4i). Finally, we examined the expression levels of FZD6 with western blot using *lncFZD6* and BRG1 silenced cells, and confirmed the positive regulation of FZD6 expression by *lncFZD6* and BRG1 (Fig. 4j).

To further confirm the role of the combination between *lncFZD6*-BRG1 and FZD6 promoter, we deleted the *lncFZD6*-BRG1 binding region of *FZD6* promoter (Fig. 4k), and found impaired combination largely inhibited liver TIC self-renewal (Fig. 4l). Altogether, *lncFZD6* interacted with BRG1 and recruited it to *FZD6* promoter, and finally drove FZD6 expression.

### WNT5A is highly expressed in liver cancer cells

**Fig. 5:**
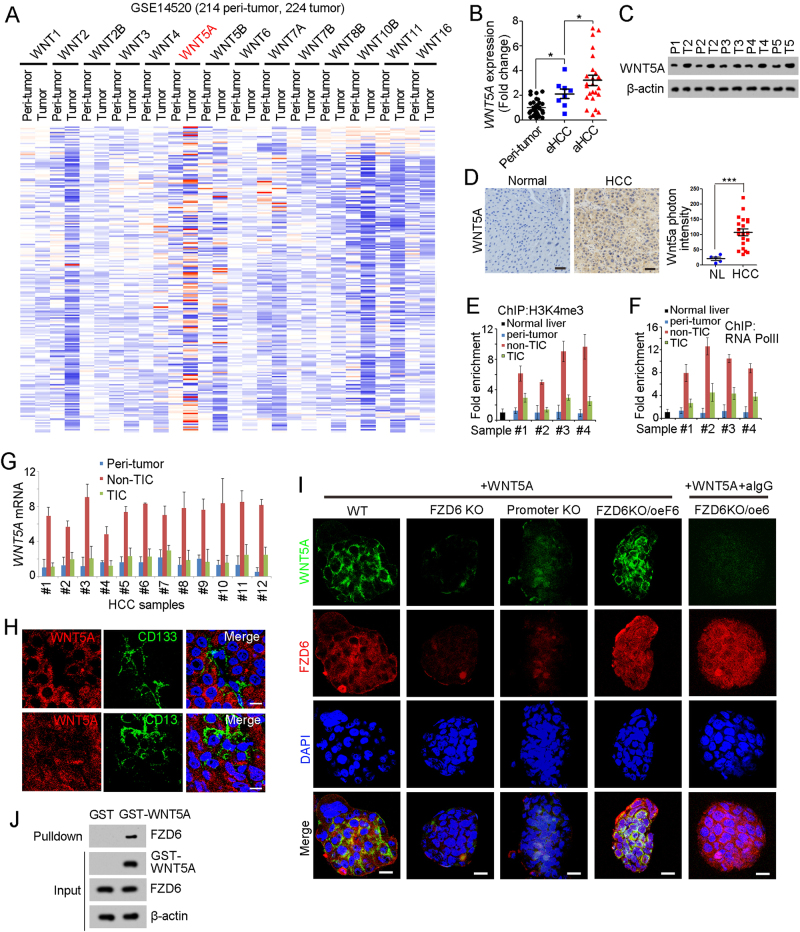
WNT5A is highly expressed in non-TICs. **a** Heatmap of indicated Wnt ligands in 214 peri-tumor samples and 224 tumor samples derived from GSE14520. The average expression levels in peri-tumor samples were defined as 1. WNT5A is highly expressed during liver tumorigenesis. **b**, **c** Peri-tumor, early hepatocellular carcinoma (eHCC) and advanced hepatocellular carcinoma (aHCC) samples were collected, and FZD6 expression levels were detected using real-time PCR **b** and western blot **c**. **d** WNT5a expression levels in normal liver and HCC samples were examined with immunohistochemistry. Typical images were shown in left panels and calculated results were shown in right. **e**, **f** The activation of *WNT5A* promoter in the indicated cells were analyzed by ChIP assays with H3K4Me3 and RNA polymerase II (RNA PolII). Normal liver cells, peri-tumor, non-TICs and TICs were sorted for ChIP assays. *WNT5A* is transcriptionally activated in normal liver tumor cells (non-TICs). **g** The indicated cells were enriched and WNT5A expression levels were analyzed using real-time PCR. **h** HCC primary samples were stained with WNT5A and CD133 (upper panel) or CD13 (lower panel), showing that WNT5A is highly expressed in CD133 and CD13 negative cells (non-TICs). Scale bars, 10 μm. **i** The indicted oncospheres were incubated with recombinant human WNT5A protein. After washing three times, the spheres were fixed with 4%PFA and permeablized with Triton X-100 buffer. The samples were stained with WNT5A and FZD6 antibodies and observed with confocal OLYMPUS FV1200. Scale bars, 10 μm. **j** GST-WNT5A was incubated with liver TIC lysate, followed by GST pulldown and western blot. FZD6 antibody was used for FZD6-WNT5A interaction. Data were shown as means ± s.d. *P < 0.05 by two-tailed Student’s *t*-test; ***P < 0.001. Data are representative of three independent experiments

We then detected FZD6 ligands in liver tumorigenesis. We analyzed WNT expression profiles using online available data, and found WNT5A is highly expressed in liver cancer (Fig. 5a). Using primary HCC samples, we confirmed the high expression of WNT5A in liver cancer, especially in advanced liver cancer (Figs. 5b-d).

We then examined transcriptional activation of *WNT5A* promoter in normal liver cells, peri-tumor cells, non-TICs and TICs, and found *WNT5A* promoter is specifically activated in non-TICs (Figs. 5e, f). High expression levels of *WNT5A* mRNA were also found in liver non-TICs (Fig. 5g). We then observed WNT5A expression profiles in primary samples, and confirmed that WNT5A is expressed in CD133^-^ or CD13^-^ non-TIC cells (Fig. 5h).

We incubated oncospheres with recombinant WNT5A and found WNT5A binds to TIC surface (Fig. 5i). What is more, *FZD6* knockout and *FZD6* promoter knockout spheres showed impaired combination with WNT5A, indicating the essential role of FZD6 in liver TIC-WNT5A attachment (Fig. 5i). Accordingly, we added FZD6 antibody and found attenuate WNT5A binding, confirming FZD6–WNT5A interaction in liver TIC surface (Fig. 5i). Through Glutathione S transferase (GST) pulldown assay, we also confirmed the interaction between WNT5A and FZD6 (Fig. 5j). Altogether, WNT5A is the predominant ligand of FZD6 and mainly expressed in non-TICs.

### WNT5A drives Wnt/β-catenin activation of TICs through *lncFZD6*-FZD6 signaling

**Fig. 6:**
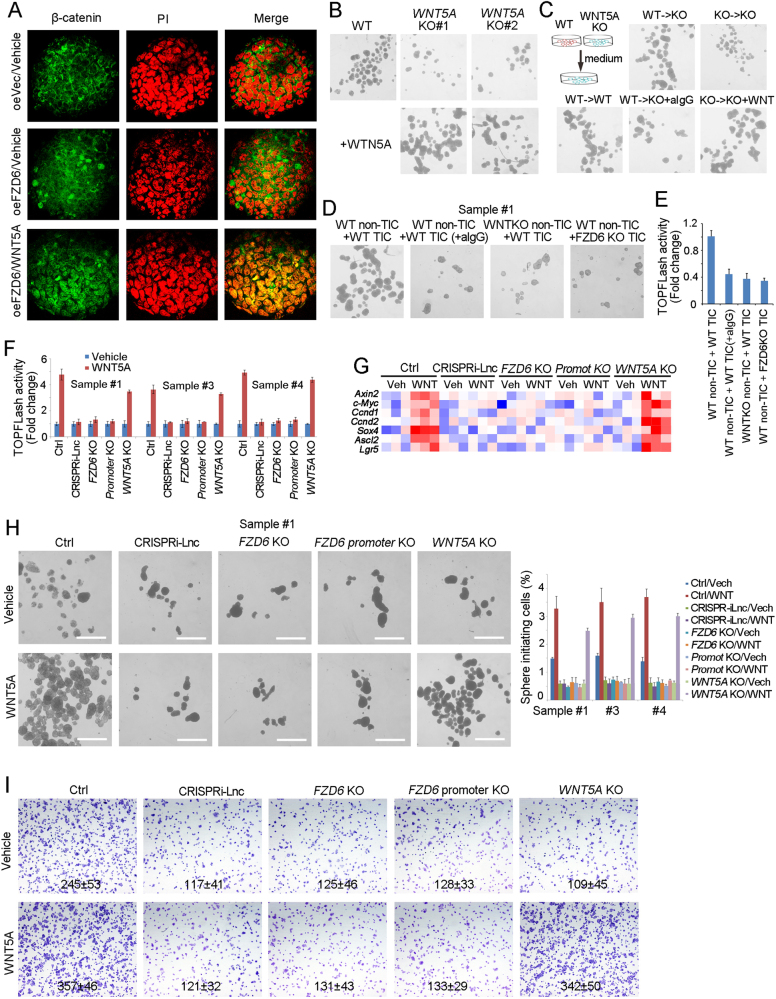
WNT5A activates Wnt/β-catenin signaling through FZD6-dependent manner. **a** The indicated treated spheres were examined for β-catenin nuclear translocation by confocal microscope. **b** Sphere formation assays were performed using *WNT5A* knockout cells, supplemented with recombinant WNT5A protein or not. **c** WT and *WNT5A* knockout cells were used for 1 week’s sphere formation and the supernatant medium were obtained for sphere formation of *WNT5A* knockout cells. Two weeks later, the typical spheres were shown. **d**, **e** WT and *WNT5A* KO non-TICs were sorted and mixed with TICs at ratio of 1:1, followed by oncosphere formation assay **d** or TOPFLash assay **e**. aIgG, WNT5A antibody IgG. **f** The indicated cells were treated with WNT5A, and Wnt/β-catenin activation was examined using TOPFLash methods. **g** The indicated cells were treated with WNT5A for 2 days, and the expression levels of Wnt/β-catenin target genes were examined by real-time PCR. The expression data were shown as heatmap. **h** In all, 1000 indicated cells were incubated in sphere formation medium with/o WNT5A for 2 weeks. Typical pictures were shown in left panels and calculated sphere-initiating ratios were shown in right panels. Scale bars, 500 μm. **i** The indicated cells were incubated with WNT5A, and tumor invasion capacity was observed with transwell assay. Typical images and invasive cell numbers (mean ± s.d.) were shown. Data were shown as means ± s.d. *P < 0.05 by two-tailed Student’s *t*-test. Data are representative of three independent experiments

We then examined the role of WNT5A in liver TIC self-renewal. First, we treated spheres with WNT5A and observed β-catenin activation by observing β-catenin subcellular location, and found WNT5A triggers β-catenin nuclear translocation and Wnt/β-catenin activation (Fig. 6a). We then found *WNT5A* knockout spheres showed impaired self-renewal capacities, and recombinant WNT5A can rescue the diminished sphere formation (Fig. 6b). We also found the medium of WT spheres promote sphere formation of WNT5A knockout cells, but WNT5A knockout medium cannot, indicating WT sphere secret WNT5A to drive self-renewal (Fig. 6c). What’s more, non-TICs could promote the self-renewal of liver TICs and Wnt/β-catenin activation, but WNT5A knockout non-TICs could not, indicating that non-TICs drove liver TIC self-renewal and Wnt/β-catenin activation through WNT5A (Figs. 6d, e).

To further explore the role of WNT5A in liver cancer, we treated primary liver cancer cells with WNT5A, and found WNT5A treated cells showed enhanced Wnt/β-catenin activation (Fig. 6f). What’s more, WNT5A treatment had impaired roles in *lncFZD6* silenced and *FZD6* knockout cells, indicating *lncFZD6*-FZD6 axis plays an essential role in WNT5A-induced Wnt/β-catenin activation (Fig. 6f). Expression profiles of Wnt/β-catenin target genes confirmed the important role of *lncFZD6* and FZD6 in Wnt/β-catenin activation (Fig. 6g). Finally, we explored the role of WNT5A in liver TIC self-renewal using sphere formation assay, and found WNT5A treatment promotes liver TIC self-renewal, and *lncFZD6*-FZD6 plays an essential role in WNT5A-induced liver TIC self-renewal (Fig. 6h). Transwell assay also confirmed the critical role of *lncFZD6*-FZD6 in WNT5A-induced tumor invasion (Fig. 6i). Altogether, WNT5A drives Wnt/β-catenin activation through *lncFZD6* and FZD6-dependent manner.

### *LncFZD6*-BRG1-FZD6 serves as a target for liver TIC elimination

**Fig. 7:**
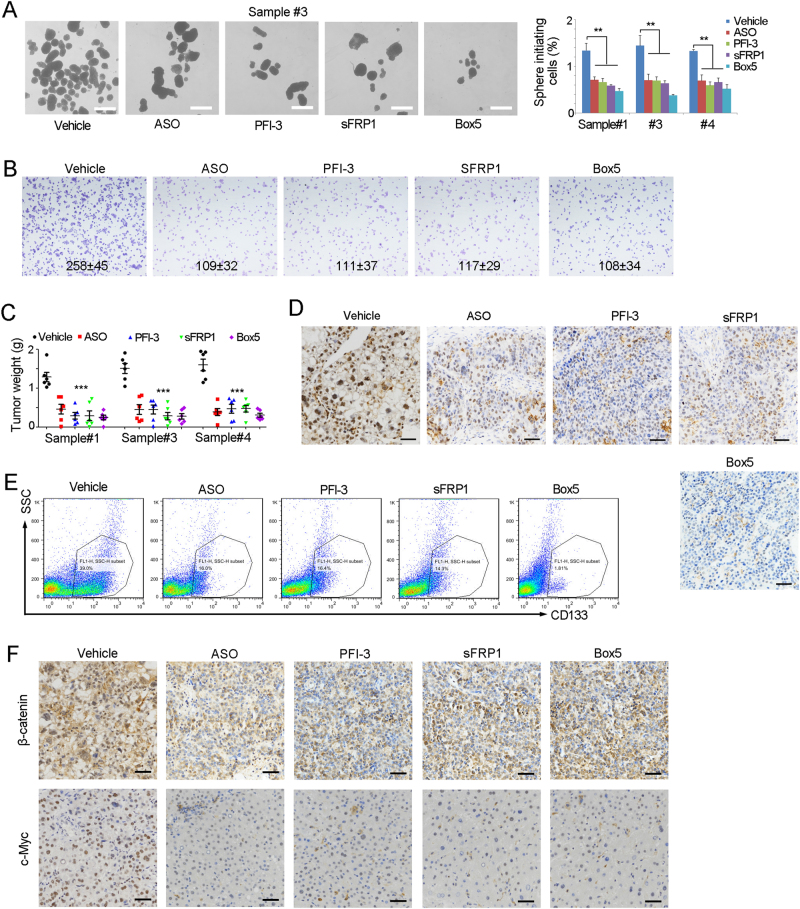
*LncFZD6*-BRG1-FZD6 can be targeted for liver TIC elimination. **a** The indicated treated cells were used for sphere formation assays. Typical images were shown in left panels and calculated ratios were shown in right panels. **b** Tumor invasion capacity of the indicated treated cells were examined by transwell assay, and typical images and cell numbers (mean ± s.d.) were shown. **c** In all, 1 × 10^6^ *lncFZD6* silenced (ASO), BRG1 inhibited (PFI-3), FZD6 antagonist (sFRP1) and WNT5A inhibitor (Box5) treated cells were injected into BALB/c nude mice for 1 month’s tumor propagation. Tumor weights were shown as scatter diagram. Six mice were used for each assay. **d** The indicated treated tumors were obtained and immunohistochemical analysis for the expression of Oct4, a functional marker of liver TICs. **e** CD133 FACS results of the indicated treated tumors. CD133^+^ liver TICs were gated and the proportion of liver TICs in tumor bulk were shown. **f** The indicated treated tumors were obtained and immunohistochemical analysis for the expression of β-catenin and c-Myc. Scale bars,** a**, 500 μm; **d**, **f**, 50 μm. For **a**, **c**, data were shown as means ± s.d. **P < 0.01; ***P < 0.001 by two-tailed Student’s *t*-test; ns not significant. Data are representative of four independent experiments

Finally, we explored the role of *lncFZD6*-BRG1-FZD6 in liver TIC elimination. We treated TICs with *lncFZD6* ASO, FZD6 antagonist, BRG1 inhibitor PFI-3, or WNT5A inhibitor, and found impaired sphere formation upon inhibition of *lncFZD6*-BRG1-FZD6 pathway (Fig. 7a). We also found the critical role of *lncFZD6*-BRG1-FZD6 in liver tumor invasion (Fig. 7b). Taking advantage of tumor propagation assays, we also found *lncFZD6*-BRG1-FZD6 pathway was required for liver TIC propagation (Fig. 7c). Then, we collected the established tumors and examined liver TICs with functional marker Oct4 and surface marker CD133. We performed immunohistochemistry assay, and found impaired expression of Oct4 in *lncFZD6*-BRG1-FZD6 silenced cells, indicating their impaired self-renewal (Fig. 7d). We also examined liver TIC population with CD133, and found *lncFZD6*-BRG1-FZD6 inhibition remarkably reduced the liver TIC numbers, again confirming that *lncFZD6*-BRG1-FZD6 can be used for liver TIC elimination (Fig. 7e). What’s more, β-catenin nuclear translocation and Wnt/β-catenin target gene (c-Myc) were impaired upon *lncFZD6*-BRG1-FZD6 blocking (Fig. 7f). Taken together, *lncFZD6*-BRG1-FZD6 blockade inhibits Wnt/β-catenin activation and eliminates liver TICs.

## Discussion

The self-renewal regulation of liver TICs is precisely regulated, and many signaling pathways are involved in liver TIC self-renewal. Wnt/β-catenin signaling, the most important signaling in liver TICs, is mainly regulated by β-catenin stability and activity [17, 18]. Upon Wnt activation, β-catenin is translocated into cell nuclei and activates the transcription of target genes [37]. The stability and transcriptional activity of β-catenin are most deeply explored; while, the initiation process of Wnt/β-catenin activation is poorly understand. It is the interaction between Wnt and Wnt receptors that initiates Wnt/β-catenin activation. Wnt molecules are secreted from “niche” cells, and Wnt receptors are highly expressed in TICs. There are many kinds of Wnt (Wnt1, Wnt2, Wnt3, Wnt3A, Wnt4, Wnt5A, Wnt5B, Wnt6, Wnt7A, Wnt7B, Wnt8A, Wnt8B, Wnt9A, Wnt10A, Wnt11, etc) and Wnt receptors (FZD1–FZD10). Here, we investigated the expression profile of Wnt receptors in tumorigenesis and liver TICs, and found FZD6 is highly expressed in liver cancer and liver TICs.

The role of FZD6 in Wnt/β-catenin signaling activation is controversial. FZD6 blocks Wnt/β-catenin activation by Wnt3a when co-expressed with FZD1 [26]. However, FZD6 is required for Wnt4 inducted Wnt/β-catenin activation [27]. We think the opposite role of FZD6 in Wnt/β-catenin activation maybe a result of different cell types and different Wnt ligands. As there is no PDZ domain-binding motif in C-terminal and the molecular mechanism of FZD6 in Wnt4 induced Wnt/β-catenin activation remains unclear [27]. Here, we examined the role of FZD6 in liver TIC self-renewal using FZD6 silenced and knockout cells, and found FZD6 is required for liver TIC self-renewal. What’s more, FZD6 serves as a target for liver TIC elimination. Using various functional assays, we found FZD6 plays a positive role in liver tumorigenesis and TIC self-renewal, indicating its positive regulation of FZD6 in Wnt/β-catenin activation along with tumorigenesis.

LncRNAs plays various roles in physiological and pathological processes [38]. Divergent lncRNAs, transcribed in the opposite direction to nearby protein-coding genes, are about 20% of total lncRNAs [39]. Divergent lncRNAs often co-expresses with its nearby genes, and regulate its nearby gene in cis [39]. Here we found *lncFZD6* is a divergent lncRNA to FZD6 gene. *LncFZD6* is co-expressed with FZD6 and regulate the expression of FZD6 through BRG1-mediated chromatin remodeling. There are several lncRNAs involved in Wnt/β-catenin activation. A recent work showed lnc β-catm promoted the interaction between β-catenin and EZH2 to drive the methylation of β-catenin, and finally inhibited β-catenin ubiquitinoylation and activated Wnt/β-catenin activation [40]. Here we found another lncRNA involved in Wnt receptor expression.

BRG1, a core component of SWI/SNF complex, plays critical role in tumorigenesis of many tumor types [41]. There are large amount of similarity (>75%) of BRG1 and its analog, BRM. Both BRG1 and BRM can form SWI/SNF complex in exclusive manner [42]. Accordingly, SWI/SNF can be grouped to BRG1-embedded SWI/SNF complex and BRM-embedded SWI/SNF complex. Recently, a work discovered that BRG1-BRM switch occurred in liver tumorigenesis, and to a large extent, the switch accounted for liver TIC self-renewal [14]. Here we found BRG1 is required for liver TIC self-renewal. BRG1 was recruited to *FZD6* promoter by *lncFZD6*. *LncFZD6*-BRG1-FZD6 can be used for liver TIC elimination.

β-Catenin mutation has been discovered as a driver mutation in subpopulation of HCC patients in clinic, and here we found another driven gene. Thus, we compared β-catenin mutation-driven HCC tumorigenesis and FZD6-driven HCC tumorigenesis. We found no FZD6 expression changes in β-catenin mutant HCC samples (Supplementary Figure 1F), and β-catenin mutation (T41A) largely impaired the role of FZD6 (Supplementary Figure 1G, H). These results indicated that FZD6-initiated signaling was upstream of β-catenin and FZD6 functions through β-catenin signaling. The detailed crosstalk needs to be further explored.

## Materials and methods

### Cells and samples

293T cells (ATCC CRL-3216) and HCC cell line PLC (ATCC CRL-8024) were obtained from ATCC. Cells were cultured in Dulbecco’s modified Eagle’s medium (DMEM) medium (GIBCO), supplemented with 100 μg/ml penicillin, 100 U streptomycin and 15% fetal bovine serum (FBS; Invitrogen).

Human liver cancer clinical samples were obtained from the first affiliated hospital of Zhengzhou University with informed consent, according to the Institutional Review Board approval. All human sample and mouse experiments were approved by the Institutional Committee of Zhengzhou University. HCC samples were ranked according to the obtained time, and several samples with sphere formation capacity (#1, #3 and #4) were used for sphere formation. The details for these sample were: #1, advanced HCC, 58 years old, male, tumor size, 7.8 × 5.2 × 4.9 mm, non-metastasis; #3, advanced HCC, 71 years old, male, tumor size, 8.2 × 4.3 × 3.2 mm, non-metastasis; #4, advanced HCC, 65 years old, female, tumor size, 5.8 × 5.2 × 4.6 mm, non-metastasis.

### Antibodies and reagents

DAPI (cat. no. 28718-90-3) and anti-β-actin antibody (cat. no. A1978) were purchased from Sigma-Aldrich. Anti-FZD6 (cat. no. HPA017991) antibody was from Atlas antibodies, anti-RNA polymerase II (cat. no. GWB-3F12B0) antibody was from GenWay. Anti-Oct4 (cat. no. 2750), anti-HeK4me3 (cat. no. 9727), anti-H3K27Ac (cat. no. 8173) antibodies were from Cell Signaling Technology. Anti-BRG1 (sc-17796) antibody was purchased from Santa Cruz Biotechnology. Phycoerythrin (PE)-conjugated CD133 (cat. no. 130098826) was from MiltenyiBiotec. Alexa594-conjugated donkey anti-rabbit IgG and Alexa488-conjugated donkey anti-mouse IgG antibodies were from Molecular Probes. T7 RNA polymerase (cat. no. 10881767001) and Biotin RNA Labeling Mix (cat. no. 11685597910) were obtained from Roche Life Science. The LightShift Chemiluminescent RNA EMSA kit (cat. no. 20158) and Chemiluminescent Nucleic Acid Detection Module (cat. no. 89880) were purchased from Thermo Scientific.

### Sphere formation

For sphere formation assay, 5000 primary HCC cells were seeded into Ultra Low Attachment six-well plates and cultured in DMEM/F12 medium supplemented with N2, B27, 20 ng/ml EGF and 20 ng/ml bFGF for 2 weeks’ incubation. The materials needed for sphere formation were: ultra low attachment plates (Corning, cat. no. 3471); N2 supplement (Life Technologies, cat. no. 17502-048), B27 (Life Technologies, cat. no. 17504-044), epidermal growth factor (EGF) (Life Technologies,cat. no. E5036-200UG) and basic fibroblast growth factor (bFGF) (Millipore, cat. no. GF446-50UG).

### Real-time PCR

Tissue RNA was extracted by TRIZOL method according to the Life’s manual. Briefly, 1 ml TriZol reagent was added into samples for 5-min incubation, followed by addition of 200 μl chloroform and then the samples were separated by centrifugation. The supernatant was incubated with isopropanol and washed with 75% ethanol. Finally, the RNA samples were dissolved with RNase-free H_2_O and used as template for reverse transcription-PCR (RT-PCR). RT-PCR was performed with RT-PCR kit derived from Promega Company, and real-time PCR was performed by standard procedures.

### Western blot

HCC samples or oncospheres were crushed within RIPA buffer (150 mM NaCl, 0.5% sodium deoxycholate, 0.1% sodium dodecyl sulfate (SDS), 1 mM EDTA, 1% NP-40 and 50 mM Tris, pH 8.0), followed by SDS–polyacrylamide gel electrophoresis (PAGE) for separation. The samples in the polyacrylamide gel were transferred to nitrocellulose (NC) membrane (Beyotime Biotechnology), and then incubated with primary antibodies and horseradish peroxidase (HRP)-conjugated secondary antibodies.

### Immunohistochemistry

In all, 5-μm formalin-fixed sections were deparaffinized and rehydrated with xylene and graded alcohols. After 15-min incubation with 3% hydrogen peroxide (H_2_O_2_), the slides were boiled in Tris-EDTA buffer (10 mM, pH 8.0) for antigen retrieval. Then, the samples were incubated in primary antibodies and HRP-conjugated secondary antibodies. After detection with HRP substrate, the samples were counterstained with hematoxylin, followed by dehydration in graded alcohols and xylene.

### Tumor propagation and initiating assay

For tumor propagation detection, 1 × 10^6^ *lncFZD6* silenced, overexpressed and control cells were injected 6-week-old BALB/c nude. The mice were sacrificed 1 month later, and tumor weight was detected. For tumor-initiating assays, 10, 1 × 10^2^, 1 × 10^3^, 1 × 10^4^, and 1 × 10^5^ *lncFZD6* silenced cells were injected 6-week-old BALB/c nude for 3 months’ tumor formation. The ratios of tumor-free mice were calculated 3 months later. For each sample of tumor propagation and tumor initiation, six mice were used.

### Transwell invasion assay

For transwell invasion assays, 3 × 10^5^ HCC cells were plated on the top chamber with Matrigel-coated membrane, and incubated in medium without FBS. Medium supplemented with FBS was added in the lower chamber as a chemoattractant. The plate was incubated in incubator for 36 h and cells that did invade through the membrane were removed by a cotton swab. Cells on the lower surface of the membrane were fixed with methanol and stained with crystal violet. The images were taken with Nikon-EclipseTi microscopy.

### Chromosome immunoprecipitation

ChIP assays were performed according to the manual of Upstate Biotechnology. Briefly, *lncFZD6* silenced spheres or wide type spheres were incubated in 1% formaldehyde at 37 °C for crosslinking, followed by treatment with SDS lysis buffer and subsequent ultrasonic for shearing DNA. BRG1, H3K4Me3, H3K27Ac and RNA polymerase II and control antibodies was added into samples for DNA segment enrichment.

### RNA pulldown

For RNA pulldown assays, biotin-labeled *lncFZD6* and control RNA were prepared in vitro with biotin RNA labeling mix (Roche). Then, the labeled RNA transcript was incubated with sphere lysate. Streptavidin beads were added and the enriched components were analyzed by SDS–PAGE and western blot or mass spectra.

### RNA immunoprecipitation

For RIP, liver TIC oncospheres were treated with 1% formaldehyde for crosslinking, and then lyzed with RNase-free RIPA buffer supplemented. The supernatants were incubated with BRG1 or control IgG antibodies and total RNA was extracted from the eluent. *LncFZD6* or control *ACTB* enrichment was detected using real-time PCR.

### Statistical analysis

Two-tailed Student’s *t*-tests were used for statistical analysis. *P* < 0.05 was considered to be statistically significant.

## Electronic supplementary material

Supplementary Figure 1
