# Supplementary material for: LncFZD6 initiates Wnt/β-catenin and liver TIC self-renewal through BRG1-mediated FZD6 transcriptional activation
Source: Oncogene. 2018 Mar 14;37(23):3098–112. doi: 10.1038/s41388-018-0203-6 (PMC5992127; doi:10.1038/s41388-018-0203-6)
Supplement: Supplementary file 1 — Supplementary Figure 1 [file 41388_2018_203_MOESM1_ESM.docx]

**
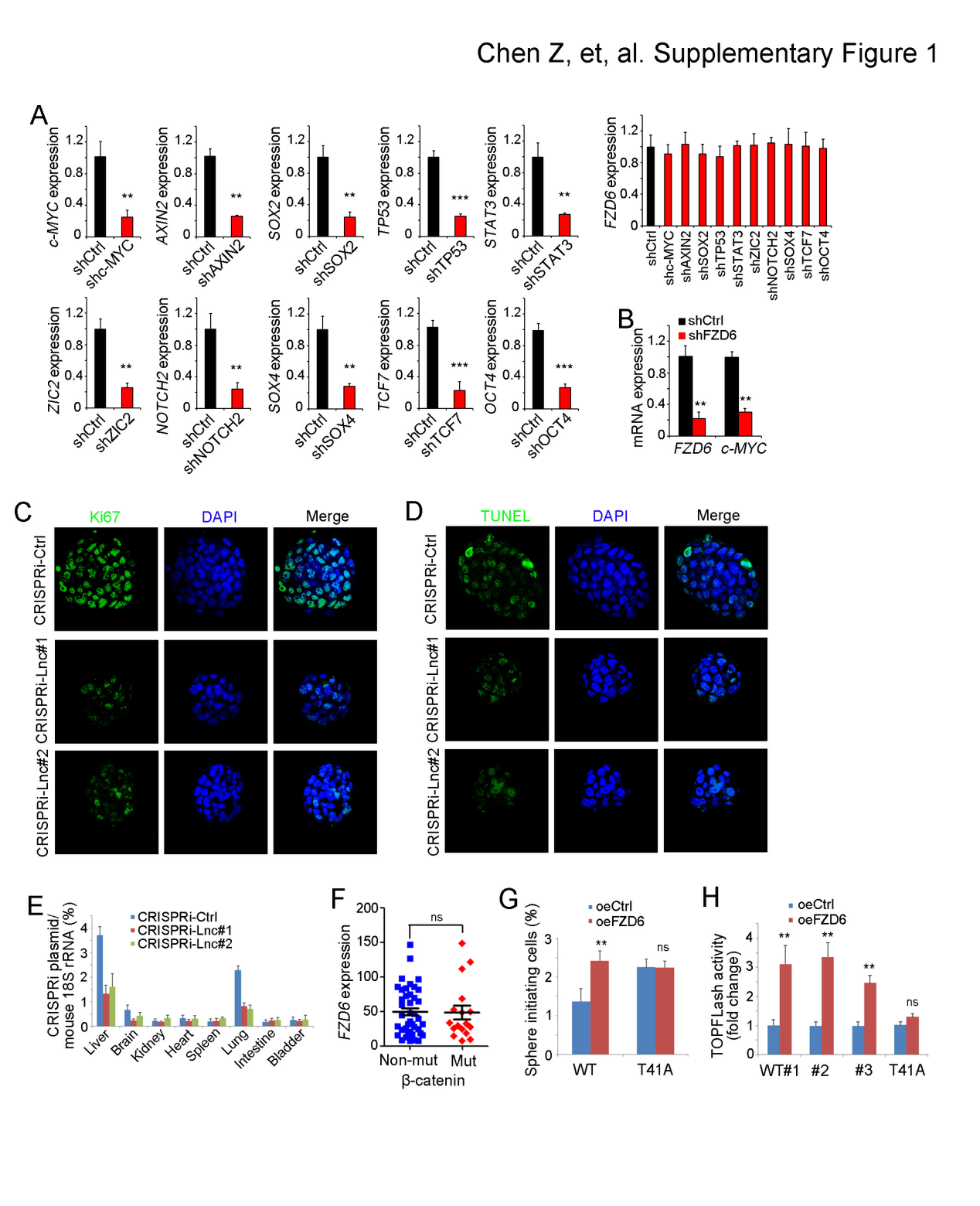
**

**Supplementary Figure 1.** (A) Primary HCC sample cells were infected with PsiCoR shRNA lentivirus targeting the indicated HCC/TIC-related molecules, followed by FZD6 mRNA examination. The knockdown efficiency was shown in left panel and FZD6 expression was shown in right panel. (B) FZD6 knockdown primary cells were generated by PsiCoR shRNA lentivirus and realtime PCR assays were performed to detect the expression levels of FZD6 and c-MYC. (C, D) LncFZD6 depleted oncospheres were generated through CRISPRi strategy. Their proliferation and apoptosis were examined by Ki67 staining (C) and TUNEL assays (D) (transferase-mediated deoxyuridine triphosphate-biotin nick end labeling), respectively. (E) CRISPRi stable expressed liver cancer cells were generated and subcutaneously injected into BALB/c nude mice. 1 month later, the tissue distribution profiles of CRISPRi plasmids were examined through realtime PCR. (F) The samples of online-available HCC dataset (E-TABM-36) were divided into two groups (β-catenin non-mut and β-catenin-mut), and expression levels of FZD6 was shown (mean±sem). (G, H) β-catenin mutation was examined by PCR and DNA sequencing, and one β-catenin mutant HCC sample (T41A) were found. HCC samples with WT-β-catenin (WT) and mutant-β-catenin (T41A) were infected with PBPLV lentivirus for FZD6 overexpression, followed by sphere formation (G) and TOPFLash examination (H). Data are representative of three independent experiments.
